# Americans Abroad

**Published:** 1870-10

**Authors:** 


### HALL'S

### JOURNAL OF HEALTH.

[*All Articles are written by the Editor.*]

VOL. XVII.—OCTOBER, 1870.—NO. X.

### AMERICANS ABROAD.

To be young, and to have abundant means to travel abroad without feeling skimped as to money matters—having plenty of time, good nature and funds—is certainly one of the greatest delights of life. The object and the manner of foreign travel are various ; fortunate are they who have the intelligence to combine the two so as to result in the highest advantage.

A gentleman had a son of sixteen who was small of his age, delicate in his organization and constitution, and withal had been so constantly at school since seven years old, that he seemed to be a runt ; he was growing stoop-shouldered ; on the instant of sitting down, his back would bend, with head forwards, as if he were a hundred years old. He seemed never to be ready to get up in the mornings, and on Saturdays and Sundays, when there was no school, he would sleep until ten and even twelve o'clock, if not waked up. His father would not allow him to be waked. It seemed to indicate that the excitement of school lessons during the week made such an excessive draft on the nervous system, that nature demanded time for making up for the over-work of the brain.

It was determined to break up such a habit of life at once, on the ground that it was better for a child to have full growth of body, and constitution, and health, than to to be a Solomon ; for it is easier to get a good education, after securing a vigorous constitution, than to secure health when once lost in acquiring an education. So the youth was sent to Heidelberg, in Germany, to eat and sleep, and travel on foot up its mountains, and over and around its castles. Letters began to come, complaining that the boy was losing time ; that he was always hungry, and did not get up until ten o'clock, and then wanted to do nothing but walk about the streets and go with the girls. To these letters the father replied, "Let him eat, and sleep, and spark ; the young ladies will mend his manners, the eating will help him grow, and as for sleep, let him get all he wants." Sometimes the father had some compunctions about his son's losing time ; "doing nothing" for two years seemed ruinous, but being backed by the principles inculcated in *Hall's Journal of Health*, he bravely determined to carry out his programme. At the end of two years the animal progress has been encouraging. When asked his opinion of Dresden, he replied, "The picture galleries are horrid, but the beefsteaks are elegant." He has grown some four inches, is as straight as a ramrod, has improved greatly in manners and morals, and is a great admirer of the ladies ; and now, with high health and a manly form and mien, wants to come home to New York, go into business, and make a living for himself.

FOR HEALTH.

Others go abroad of their own accord for health, for observation and enjoyment. A young lady writes from the mountains of Saxon-Switzerland, a very lovely part of Germany :

"Schandau, where we are now, is a central point, from which we can make excursions in all directions, and thus see all the country. The children have joined us. Their appetites are perfectly astounding. They seem never to have enough, and are always asking for more. The country around is lovely. We rejoice in the magnificent name of 'Palais Royale.' M. don't seem to care about anything but to get enough to eat. There are many ways of relieving travelers of their money. As soon as a stranger takes possession of his room, the 'Band' comes and gives a serenade, for which at least a dollar has to be paid. Then every person has to pay a dollar and a half for the laying out of the walks and drives, which are very beautiful.

"Schandau is quite a fashionable watering-place with the Germans. The other day we went on an excursion with our German teacher, Dr. P., and a young Englishman, and returned to tea. Next Thursday we are going again, and shall be gone all day.

"Saxon-Switzerland is a small part of Saxony, and is quite renowned for its beauty, and I suppose resembles the real Switzerland. The country around is very beautiful, the chief object of interest being the curiously shaped rocks, which are very large ; some of them resemble fortresses ; on clear days they can be seen from Dresden, two hours away.

"Schandau is a small town, situated in the heart of a perfect little valley, with a beautiful stream of mineral water running the whole length. Sitting on our balcony, we see nothing but hills. The house itself is on the side of one ; in front there is a broad carriage road ; the stream is beyond that, and the hills on the other side covered with evergreens, and both ends of the valley, which is about ten miles long, are closed in with the same. The whole valley is beautifully laid out with walks, and travelers are taxed to keep them in repair. I, no doubt, shall get my money's worth. M. has a perfect horror of climbing hills ; it is quite a joke among us, and whenever we see a particularly high point we always propose mounting it. Our excursion of next Thursday requires four hours' walking, a great portion of which will be ascending, as we are going to three of the highest points in Saxon-Switzerland—Kunstall (Cowstail) is the name of one—Thunterberg.

(Thunderhill) another; the third I do not remember. We are very foitunate in having Professor Verchel for a guide through the country, for he knows it well."

These items have been introduced as hints to excursionists, whether in our own country or in foreign lands. To make your traveling available at all points, make it your business to acquaint yourself with the localities, with all the objects of interest far and near; visit them on foot if practicable, mount every hill-top, dive down into every valley, explore every cave, cross every stream, gather every flower, examine every pebble; talk with the people; notice their ways of living, observe all their peculiarities, and be out of doors every hour of daylight that is possible. In this way health is promoted, information gained, and pleasures multiplied, besides laying in a stock of ideas, and facts and incidents, with which you may interest and delight friends in their recountal as long as you live, besides being an unfailing source of pleasurable reminiscence to yourself in all after life, liable to flit across the mind as often as you take up a newspaper or peruse a magazine, at the sight of a name or place which you have once visited.

We were once at Quebec; a party of ladies and gentlemen came in late at night from the States, and on inviting them to go on an excursion next morning to some remarkable places a few miles in the country, they plead "haste," as they were going to return early in the morning, and had just come there in order to have it said they had been to Canada, and had seen a walled city.

Dr. C., who had spent a number of years in foreign travel, especially in Arabia, Syria, and across the desert in caravans, said the other day that he was greatly amused at a gentleman and lady who had arrived at Cairo or Jerusalem—we don't remember which—and were going out to see the place after supper, intending to leave for home next morning.

It is a common thing, wherever we go in summer, to see young ladies loaded with magazines and novels ; and whether in cars, or on canal boats, or in country coaches, or at hotels, their noses are forever stuck in a book of some sort, instead of looking about them, getting views of the beauties of the country, its nature, its peculiarities, its charms and its capabilities. We had a great deal better be at home reading and loafing around the house, for then at least the price of room and travel would be saved.

As for young men—students, clerks and other sedentary persons—the happiest and most healthful mode of making a summer excursion is to go in congenial parties of half a dozen, travel on foot, and camp out in the woods or among the mountains, and thus breathe an out-door air, during the whole of twenty-four hours ; there would be an enjoyment and an improvement in health, and strength and digestion more decided in ten days, than in a month's time hanging round a hotel or boarding-house.

